# How Do Retinoids Affect Alzheimer’s Disease and Can They Be Novel Drug Candidates?

**DOI:** 10.7759/cureus.57548

**Published:** 2024-04-03

**Authors:** Mazen Alsharief

**Affiliations:** 1 International Postgraduate Medical Training Scheme (IPGMTS), University Hospitals Birmingham, Birmingham, GBR

**Keywords:** medicinal chemistry, retinoic acid, tamibarotene, alzheimer's disease, drug design, drug discovery

## Abstract

Alzheimer’s disease is a chronic, neurological condition that faces many challenges in its management and therapy nowadays highlighting the importance and urgent need of researching new ways of approaching this disease. Retinoic acid and its derivatives, collectively known as the retinoids, are considered promising agents that have disease-modifying properties in affecting Alzheimer’s disease. This thesis aims to address the research questions of what the role of retinoids is in Alzheimer’s disease, and whether they can be used as a novel drug candidate for treating this condition.

Retinoids’ properties and agonistic actions on the nuclear receptors retinoic acid receptor (RAR) and retinoic X receptor (RXR) affect various pathways as well as their underlying genetic factors that compose important pathophysiological hallmarks causing the progression of Alzheimer’s disease as amyloid β (Aβ) production and deposition, neurofibrillary tangle (NFT) formation and phosphorylation, and inflammatory and autoimmune responses. Retinoic acid inhibits the amplification of these pathways and modifies the disease progression in animal models, proposing a solid basis for human trials. Hence, investigating retinoids as pharmacological agents in human trials has been conducted, and several synthetic analogues have been developed to address issues concerning retinoic acid’s instability and short half-life, as well as adverse drug reactions. The most prominent of these analogues is tamibarotene, a stable retinoic acid derivative with a higher half-life, higher specificity to target receptors, and fewer adverse reactions.

A number of criteria that explain what a novel drug candidate should have when managing Alzheimer’s disease have been formulated, and which also explain why most novel drug candidates other than retinoic acid have failed in achieving clinical results. Most of these candidates share one common trait which is a single-target approach in targeting disease pathways. This means that when administering these agents, their actions are to target a single disease-causing pathway at a time but do not affect other pathways. On the other hand, tamibarotene is a novel drug candidate that targets a range of pathways at once and provides a more comprehensive approach in its pharmacological actions.

## Introduction and background

**Figure 1:**
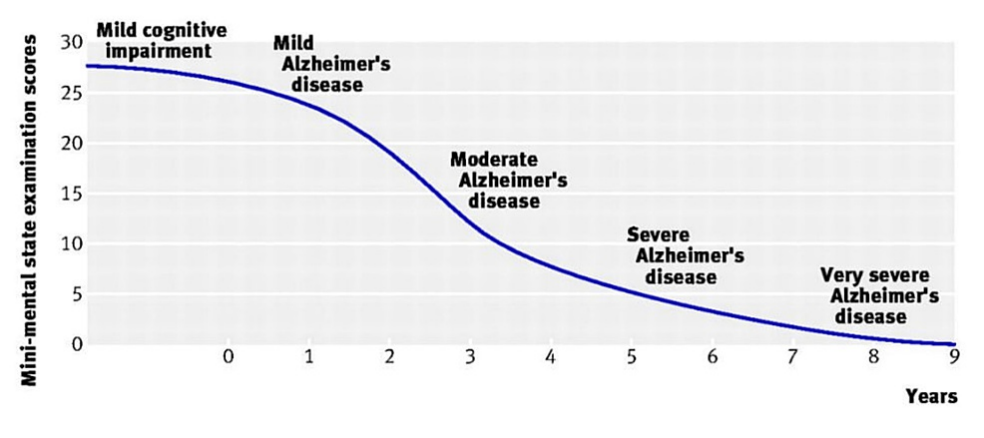
Mini Mental State Examination Scores Alzheimer’s disease progression and noticed deterioration in condition over an average time span of nine years. Figure reprinted with permission from Burns and Iliffe, 2009 [1].

Alzheimer’s disease is a chronic, progressive neurodegenerative disorder that has been the subject of great focus and importance in both academic research as well as in clinical practice. It is characterized by three main types of symptoms: cognitive dysfunction, psychiatric symptoms, and basic and instrumental loss of function in daily life activities [1]. The underlying relevance and importance of Alzheimer’s disease in research is not merely a result of its unidentified cause [1], but rather due to its great epidemiological impact represented in its prevalence. Over 36.5 million people are affected by dementia worldwide of which the majority has Alzheimer’s disease, in addition to an estimated 5-7 million new cases of Alzheimer’s disease that are identified on an annual basis [2]. No doubt, this inflicting condition imposes a great burden not just on those affected, but on related family and caregivers as well whether home-based, in elderly care facilities, or in hospitals. Also, the financial burden inflicted by dementia generally and Alzheimer’s in particular, cannot be taken lightly. In 2005, societal costs of dementia approximated 240 billion British pounds [2]. Clinically, Alzheimer’s disease can be classified according to the status of disease progression. Figure 1 illustrates the clinical stages and manifestations of Alzheimer’s disease.

Mild cognitive impairment includes signs and symptoms of memory loss but no conclusive evidence of Alzheimer’s disease. Mild Alzheimer’s disease shows increased forgetfulness and memory loss, repetitive questions and a mild impairment of daily living activities. Moderate Alzheimer’s disease shows an advancement of the previous signs and/or symptoms with emergence of dementia, which is a term that describes diminishing mental processes as memory-related symptoms, impaired reasoning and judgement, and personality changes. Severe Alzheimer’s disease portrays more severe dementia and a definite need of assistance in feeding, dressing and other daily activities. Very severe Alzheimer’s disease characterizes itself with loss of speech and basic psychomotor skills necessary for basic daily activities. There are several risk factors and disease pathways that are related to Alzheimer’s disease. The major risk factors are old age and obesity. Indirect risk factors include diabetes, hypertension, stress and raised glucocorticoid levels. Amyloid β (Aβ) plaque accumulation and the underlying genetic factors to that, tau and its intracellular tangles, apolipoproteins and its relation to lipid-protein homeostasis in the brain, and immune-dysfunction are the most prominent pathways believed to cause the disease [2].

Vitamin A (retinol) and vitamin A derivatives (retinoids) are compounds of diverse biological functions. Their actions affect various physiologic properties and influence major pathways such as embryonic development, cell growth, differentiation, and apoptosis, as well as playing a role in the central nervous system [3]. It is important to distinguish certain overlapping nomenclatures at this point and that is achieved by understanding what the collective term “retinoids” refers to. Any compound that is structurally related to vitamin A is referred to as a retinoid, including all-trans-retinoic acid (ATRA) and its analogs, and 9-cis retinoic acid (9cRA) [4]. Retinoic acid receptors (RARs) and retinoic X receptors (RXRs) are nuclear receptors that belong to a superfamily of various transcription factors that have the ability to directly bind to deoxyribonucleic acid (DNA) and modify gene expression, and are divided into nuclear hormone receptors and orphan nuclear receptors. Orphan nuclear receptors, as RXRs, derive their nomenclature from the uncertainty as to what ligand in particular binds to them at the time of identification [4,5]. Retinoids bind to RARs and RXRs, thereby exerting nuclear receptor functions. ATRA and its analogues bind to RARs, and the vitamin A derivative 9cRA binds to RXRs [4]. As RARs and RXRs accept the binding of ligands, they form a ligand-dependent transcriptional regulator complex that binds to a specific retinoic acid response element (RARE) present on the promoter region of target retinoid genes [6]. Orphan nuclear receptors, as RXRs, derive their nomenclature from the uncertainty as to what ligand in particular binds to them at the time of identification [4,5]. The most prominent retinoid is vitamin A’s active metabolite, retinoic acid. Retinoic acid plays a potential role in modulating Aβ plaque aggregation, tau hyperphosphorylation, inflammation and oxidative stress, and RAR signalling pathways and neurotransmission, all of which are altered in Alzheimer’s disease and are correlated to the development and progression of the disease [3]. Aβ peptide is a natural metabolic product that results from proteolysis of amyloid β precursor protein (AβPP) via the enzymatic actions of β-site amyloid precursor protein cleaving enzyme 1 (BACE1), β-secretase, and γ-secretase, which form a protein complex containing catalytic presenilin 1 (PS1) [3].

Aims

The aims of this thesis are to study how retinoids affect Alzheimer’s disease pathways, what mechanisms of actions they have in altering the disease progression, and to answer the question of whether they can be used as a novel drug candidate in treating Alzheimer’s disease. In order to achieve this, this thesis will investigate retinoids and their effects on Alzheimer’s disease-associated genes, their effects on inflammatory and autoimmune pathways related to Alzheimer’s disease, their effects on tau tangles and Aβ aggregates, and pharmacological aspects relevant to assessing the possibility of being a novel drug candidate.

## Review

Retinoids and their effect on Alzheimer’s disease-associated genes

A number of genes have been identified that are affected by retinoic acid and are also related to Alzheimer’s disease pathways. Zn-superoxide dismutase (SOD-1), which is mainly related to amyotrophic lateral sclerosis, and Mn-superoxide dismutase (SOD-2) gene expression are induced by ATRA and are mitochondrial-localized antioxidant enzymes [7]. Mitochondrial antioxidant function induced by ATRA is reported to protect embryonic neurons from oxidative damage by inhibition of glutathione depletion [8]. Moreover, ATRA-related SOD-1 and SOD-2 gene expression results in reduced staurosporin-induced oxidative stress and apoptosis in primary hippocampal cultures [9]. These functions are neuroprotective against oxidative, mitochondrial damage that relate to Alzheimer’s disease progression [10]. Furthermore, 9cRA up-regulates adenosine triphosphate (ATP)-binding cassette transporter A1 (ABCA1) expression. This gene affects cellular, peripheral and apolipoprotein A1 (APOA1)-mediated cholesterol efflux leading to decreased intracellular cholesterol levels causing the reduction of β and γ-secretase activities. The role of cholesterol and its metabolism throughout the central nervous system has been brought in correlation with Alzheimer’s disease progression, but it is unclear whether the correlation is of causative nature or rather a consequence of the disease’s pathology. The stronger hypothesis inclines toward it; cholesterol having a more crucial role in Alzheimer’s disease rather than it merely being a consequence. This is strengthened by cholesterol’s role in the formation of lipidated apolipoproteins APOA1 and apolipoprotein E (APOE) that promote the clearance of Aβ [11]. Another factor strengthening cholesterol’s causative correlative nature to Alzheimer’s disease is found in a link between cholesterol levels and amyloid precursor protein (APP) metabolism. This is due to the identification of α-secretase being affected by cholesterol. This is shown in a study that induced inhibition of cholesterol formation in neural cell lines which caused increased α-secretase secretion that, contrary to β and γ secretase, does not produce pathologic Aβ from APP cleavage [12]. The relevance of this in Alzheimer’s disease progression is by the reduction of Aβ and amyloid γ (Aγ) protein precursor stability and ultimately the decrease of Aβ and Aγ production that results from the reduced β and γ-secretase activities and increased α-secretase activity [13]. Retinoic acid also induces arachidonic acid release by RAR-mediated activation of the phospholipase A2 (PLA2) and phospholipase C (PLC)/diacylglycerol lipase pathways, which play a crucial role in cell proliferation, differentiation, and apoptosis. Moreover, arachidonic acid and its metabolites influence neurite outgrowth as well as neurotransmitter release. Retinoic acid receptor complexes bound to PLA2, C, and D, affect the redistribution of arachidonic acid in neuronal membranes during differentiation and growth suppression phases. In Alzheimer’s disease, impaired retinoid metabolism affects the downstream transcriptional regulation of PLA2-mediated signal transduction, and hence impairs the above-mentioned neuro-physiological properties related to PLA2 and retinoic acid [14]. Moreover, ATRA regulates Aβ peptide levels by upregulating the expression and activity of α-secretase, inhibition of β-secretase and γ-secretase, or by both together, resulting in Aβ peptide level regulation by promoting the formation of non-harmful Aβ peptides instead [15]. In addition, ATRA downregulates BACE1 expression in the brains of Tg2576 mice which are one of the most well characterized, and widely used, mouse models of Alzheimer’s disease [16].

Retinoids’ effects on tau tangles and amyloid β aggregates

**Figure 2:**
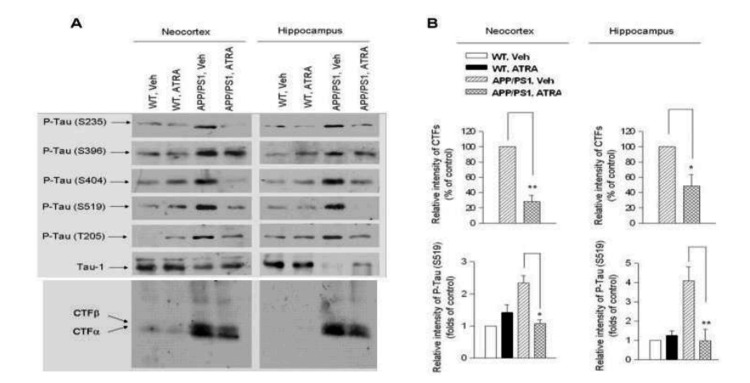
Western Blot Analysis A: Western blot analysis using anti-CTF antibody comparing the effects of ATRA-treated APP/PS1 mice to vehicle or wild-type (WT) littermates in neocortex and hippocampus samples. APP–CTF production was decreased in ATRA-treated APP/PS1 mice compared to the vehicle APP/PS1 mice. In addition, the figure also shows levels of tau phosphorylation which is decreased at Ser235, Ser404, Ser519, and Thr205 in both the frontal cortex and hippocampus of the ATRA-treated APP/PS1 mice, whereas S396 and T668 have not shown a significant decrease. Tau 1, which is a tau antibody, levels were decreased in ATRA-treated APP/PS1 mice. B: This part of the figure shows the quantitative analysis of results by a bar-chart comparison. Relative intensity of CTFs shows APP–CTF production was decreased in ATRA-treated APP/PS1 mice compared to the vehicle APP/PS1 mice by 70% in the frontal cortex and 50% in the hippocampus. Relative intensity of tau S519 shows an approximate 50% decrease in tau phosphorylation of the frontal cortex and a 75% decrease in the hippocampus in the ATRA-treated APP/PS1 mice relative to vehicle-treated APP/PS1 mice. Data are mean ± standard error of mean (SEM) from six mice per genotype. *p < 0.05, **p < 0.01 versus vehicle-treated control APP/PS1 mice. Public-use figure reprinted from Ding et al., 2008 [19]. CTF: C-terminal fragment, ATRA: all-trans-retinoic-acid, APP: amyloid precursor protein, PS1: presenilin 1

Neurofibrillary tangles (NFTs) in Alzheimer’s disease are composed of paired helical filaments of highly phosphorylated tau protein. In accordance with Aβ aggregates, they form one of the most prominent hallmarks in Alzheimer’s disease pathophysiology. High densities of tau tangles occur in brain regions responsible for a number of cognitive functions that are affected in Alzheimer’s disease. Furthermore, in order for tau protein to impact these brain regions, it must undergo certain structural changes as truncation, confrontational changes, polymerization and hyperphosphorylation [17]. Retinoic acid affects tau tangles in a number of ways. First, genomic analysis of genes related to Alzheimer’s disease progression shows that RAR-ligand complexes regulate the expression of microtubule-associated protein tau (MAPT) promoter gene located on chromosome 17q21 via presence of a RARE [18]. Moreover, a double-blinded study conducted on APP and PS1 transgenic mice that were treated with ATRA at a duration of 8 weeks revealed a reduction in tau hyperphosphorylation. The rationale explaining this outcome relates to ATRA’s effect on Aβ deposition. This deposition occurs by APP cleavage by BACE1 enzyme at the N-terminal region which produces membrane-bound APP-C-terminal fragments (CTFs) which are early markers for Alzheimer’s disease. Western blot analysis using an anti-CTF antibody to the transgenic, ATRA-treated mice showed a significant decrease in the production of APP-CTFs which can be seen in Figure 2. Moreover, it also shows a western blot analysis using antibodies against different phosphorylation sites on tau protein revealed a decrease in phosphorylation at most of these sites in ATRA-treated transgenic mice compared to controls. Quantifying this analysis showed a decrease in tau phosphorylation by 75% in the hippocampus and 50% in the frontal cortex [19], which are areas of tau tangle deposition seen in patients with early-onset Alzheimer’s disease [20]. Furthermore, the study on the transgenic mice shows a slight increase in tau immunoreactivity in ATRA-treated mice compared to controls which may indicate an immune-modulated action interfering with tau tangle formation, stimulated by retinoic acid. Also, ATRA-treated transgenic mice have a considerable decrease in cyclin-dependent kinase 5 (CDK5) activity. CDK5 causes abnormal hyperphosphorylation of tau, and as a consequence, the downregulation of CDK5 activity results in decreased tau hyperphosphorylation [19]. ATRA may also play a role in reducing tau hyperphosphorylation by suppressing cell cycle proteins as cyclin B1 which is involved in the phosphorylation of tau and aberrantly expressed in Alzheimer’s disease patients [7]. In addition to the direct role of ATRA on tau tangle formation, it can also indirectly inhibit tau hyperphosphorylation in Alzheimer’s disease by the RARA/glycogen synthase kinase 3 beta (GSK-3β) pathway. MicroRNA (miRNAs) contribute to the disease progression of Alzheimer’s disease by miR-138, a brain-enriched miRNA, which is increased in Alzheimer’s disease. Overexpression of miR-138 leads to the activation of GSK3β by targeting RARα, resulting in tau hyperphosphorylation. Furthermore, blocking miR-138’s effect on RARα by using a RARα agonist suppresses GSK-3β activity, and reduces tau phosphorylation induced by miR-138 (Figure 2) [21].

A dysregulation in the metabolism of Aβ peptide with consequent alterations in synthesis and deposition results in aggregation and formation of extracellular amyloid plaques [3]. Disturbances in retinoic acid and retinoic acid signalling in the brain contribute to abnormal Aβ formation and deposition. This relationship can be demonstrated in a number of ways that also show retinoic acid’s effect in regulating Aβ peptide metabolism. Disruption of retinoic acid signalling was achieved in an experiment conducted on rats by inducing dietary vitamin A deprivation that lasted a year. As a result, suppression of RARα and choline acetyl transferase (ChAT), as well as a decrease in RARβ, BACE, and AβPP-CTF levels occurred, in addition to an accumulation of Aβ in the forebrain cortical neurons. Reintroducing retinoic acid restored these levels [22]. Retinoic acid also influences Aβ formation by regulating genes involved in this process. Insulin degrading enzyme (IDE) and neprilysin (NEP) are both involved in Aβ peptide degradation and clearance. IDE’s transcription is regulated by RARE, and lowered IDE mRNA levels and elevated Aβ accumulation have been observed in Alzheimer’s disease [3]. Consequently, secretion of inflammatory cytokines as tumor necrosis factor alpha (TNFα) lead to the downregulation of genes involved in the clearance of Aβ as IDE and NEP, which can be reversed by an RARα agonist as AM 580 [23]. Another way how retinoic acid signalling affects Aβ is through liver X receptors (LXRs) which are expressed in macrophages in the form of LXRα, and cause the transcriptionally modulated expression of APOE. The importance of this lies in macrophages’ ability to degrade Aβ. Macrophages expressing APOE2 allele are the most efficient at degrading Aβ, followed by APOE3, and then APOE4 [24]. It is also reported from an in vitro study that retinoic acid can dose-dependently directly inhibit Aβ aggregation, as well as degrade preformed Aβ fibrils [25]. A possible mechanism of action explaining this is due to retinoic acid’s inhibition of Aβ oligomerization, as well as its specific binding to the C-terminal portion of Aβ which prevents the aggregation of Aβ [26]. Also, ATRA is reported to prevent Aβ aggregation by affecting its dysfunctional axonal transport. The alteration in axonal transport is caused by overexpression of CDK5 activator 1 p25 that leads to increased forebrain Aβ levels, whereas ATRA reduces p35 levels and thereby prevents its cleavage into p25 attenuating Aβ accumulation [19].

Retinoids, inflammation and autoimmunity

Inflammation and inflammatory mediators play a role in the disease progress of Alzheimer’s disease. The association of certain inflammatory genes with Alzheimer’s disease, the expression of inflammatory mediators, microglial activation, and clinical studies on Alzheimer’s disease patients using long-term nonsteroidal anti-inflammatory drugs (NSAIDs), exemplify this role. It is still uncertain, however, whether inflammation plays a causative or a consequential role, or whether it’s both [27]. Furthermore, an example describing inflammation and inflammatory mediators’ role in Alzheimer’s disease is the effect of Aγ-stimulated signaling pathways with Aγ aggregate production, and their corresponding inflammatory cytokine release as interleukin (IL)-1γ, IL-6, and TNF-α, acute phase proteins, chemokines as chemokine (C-C motif) ligand 2 (CCL2), and reactive nitrogen species (RNS) and reactive oxygen species (ROS). These inflammatory mediators block the necessary phagocytosis of Aγ-aggregates resulting in more inflammatory cytokine production [3]. Retinoic acid and retinoids can potentially alter inflammatory pathways in Alzheimer’s disease in a number of ways. Firstly, retinoic acid and other retinoids such as 13-cis-retinoic acid and retinaldehyde inhibit IL-6 produced by IL-1-stimulated cells at a certain dose. Retinoic acid also decreases IL-6 mRNA accumulation and gene transcription indicating that IL-6 inhibition is transcriptionally mediated [28]. In addition to IL-6 inhibitory properties, retinoic acid receptors in the central nervous system have been investigated to study the actions of tamibarotene which is a RARα/β agonist. Tamibarotene prevented inflammatory-mediated damage on dopaminergic neurons in rat midbrain slice cultures and protected it from injury mediated by lipopolysaccharide-activated microglia. In addition, tamibarotene increased brain-derived neurotrophic factor mRNA which is a neuronal growth factor, suggesting neuroprotective properties [29]. Prostaglandins are inflammatory mediators and are synthesized by astrocytes in the central nervous system. The effect of retinoic acid on central nervous system prostaglandin production has been shown in a study that was conducted on cultured cortical astrocytes that have been stimulated by lipopolysaccharides (LPS) to induce the expression of enzymes for the production of arachidonic acid and prostaglandin (PG)E2. Retinoic acid has reduced prostaglandin biosynthesis by approximately 60% by acting on cyclooxygenase (COX-2) mRNA in astrocytes [30]. Other important inflammatory mediators believed to play a role in chronic neurodegenerative diseases as Alzheimer’s disease are TNFα and nitric oxide released by microglia. LPS in addition to Aβ peptide has been used to stimulate cultures of rat microglial cells to increase mRNA expression of TNFα by 6-116-fold, and inducible nitric oxide synthase by 8-500-fold. The administration of ATRA has been dose-regulated, and its actions have been dose-dependent at 0.1-10 microM reducing mRNA expression of TNFα levels by 29-97% and inducible nitric oxide synthase by 61-96%. Moreover, ATRA has also caused the inhibition of nuclear factor kappa (NF-κB) of beta cell translocation which controls transcription of cytokine production of B cells, showing retinoic acid’s properties in altering cytokine and inflammatory mediator production [31].

Pharmacological aspects of retinoids

At this point of the thesis, it is relevant to highlight pharmacological aspects of retinoic acid, as pharmacokinetics and different analogues that have been developed so far, and to emphasize the potential of retinoic acid and its analogues as potential drug candidates in Alzheimer’s disease. Retinoids are pharmacologically unstable compounds because they include conjugated double bonds that rapidly oxidize and/or undergo isomerization in the presence of oxidants, heat, or light [3]. This explains the short half-life (t1/2) of ATRA and 13-cis RA in both rat and human models. In a rat model, an oral dose of (2mg/kg) ATRA and an injection of 13-cis RA (2.5 mg per 360 g body weight) have a t1/2 of 0.438 ± 0.124 h and 0.72 ± 0.088 h respectively. In humans, orally administered ATRA is rapidly eliminated at t1/2 of approximately 45 minutes. A single oral dose of 80 mg 13-cis RA had a mean t1/2 of 0.5 hours [32]. In the same study, a comparison was performed between plasma pharmacokinetics and brain samples of the same animals. The brain samples were taken from white and gray matter and showed high permeability of ATRA and 13-cis retinoic into white matter with a peak concentration of 25.7 μg/g in white matter and a peak concentration of 19 μg/g in gray matter. These concentrations were approximately six to seven times higher than plasma concentrations indicating higher uptake in brain tissue. t1/2 of both ATRA and 13-cis retinoic did not differ significantly between plasma and brain samples [32].

**Figure 3:**
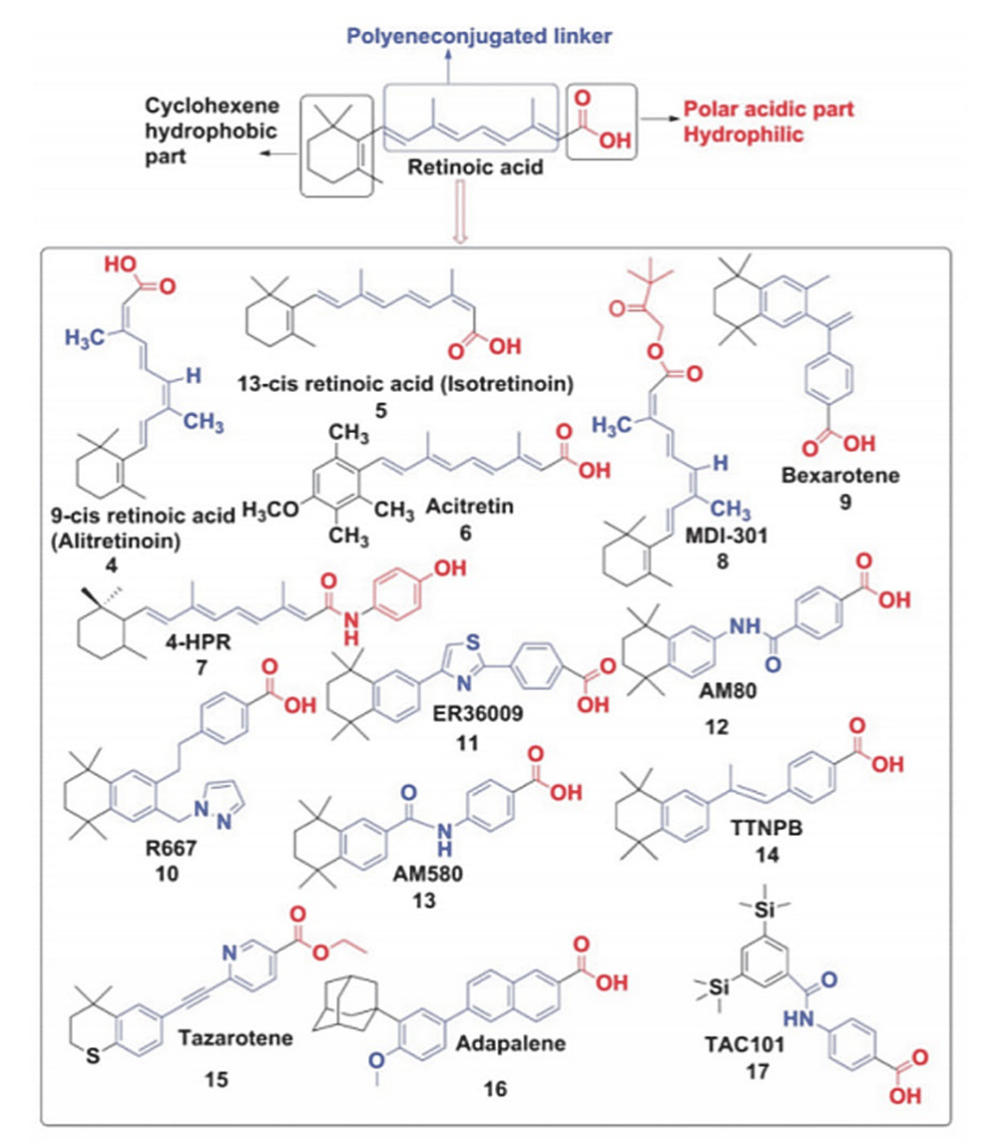
Chemical Structures The chemical structure of retinoic acid has a polar acidic part composed of carboxylic acid linked to a hydrophobic part composed of cyclohexene by a polyene-conjugated linker. This basic structure has been modified and several retinoic acid-related compounds produced. Of clinical relevance are alitretinoin (antineoplastic agent), isotretinoin (dermatological applications as acne), bexarotene (antineoplastic agent), acitretin (second-generation prescriptional retinoid used for psoriasis), and tazarotene with adapalene (both are third generation prescriptional retinoids used for acne and psoriasis). The most prominent compounds that are still used in academic research are AM80 (tamibarotene, currently only marketed in Japan as an antineoplastic agent), AM580, and TTNPB which are stable, synthetic retinoic acid derivatives used in many studies involving RARs and RXRs due to their potent agonistic actions and resistance to enzymatic metabolism. Figure reprinted with permission from Chakrabarti et al., 2015 [3]. RAR: retinoic acid receptor, RXR: retinoic X receptor

Regarding adverse reactions to ATRA administration, the following study was performed to assess its side effects. It evaluated ATRA’s pharmacokinetics by administering ATRA to 49 cancer patients at a dose range of 45 to 309 mg/m2 per day. Severe toxicities occurred with an initial peak ATRA concentration of > or = 0.5 microgram/ml (1.7 microM) and involved hypertriglyceridemia, mucocutaneous dryness and headaches [33]. Moreover, side effects resulting from 13-cis RA administration on a human model include severe headaches, urethritis, dermatitis, vertigo, and ataxia, at doses exceeding 60 mg/m2 [34]. The high toxicity of off-target binding of ATRA and 13- cis RA led to the development of many synthetic analogues using rational drug design integrated with computational modeling to produce compounds that are less metabolized by enzymes such as cytochrome P26 (cyp26) and isomerases which enable exploring the full potential of retinoids as novel pharmaceutical compounds [3]. Examples are shown in Figure 3. An interesting retinoic acid synthetic analogue that has promising potential against Alzheimer’s disease is tamibarotene. This analogue contains a carbamoyl functional group in the polyene-conjugated linker and serves to provide a more stable compound. This stability is shown as tamibarotene sustains higher plasma concentrations as opposed to ATRA’s faster declining plasma concentrations on daily administrations due to its lower affinity for cellular retinoic acid binding proteins [35]. Furthermore, tamibarotene administration to Alzheimer’s disease model mice decreased Aβ deposition, as well as having immunomodulatory effects which reduce secretion of inflammatory cytokines by astrocytes and microglia surrounding Aβ plaques [36]. Clinically, the use of tamibarotene is approved in Japan for refractory acute promyelocytic leukemia. This enabled post-marketing surveillance of adverse reactions. The most common of them are reversible triglyceride elevation, bone pain, and skin rashes at a much milder level than rashes developed by the use of ATRA (Figure 3) [35].

Discussion

In theory, retinoic acid is expected to deliver promising results when used to treat Alzheimer’s disease. However, with the number of synthetic analogues present, the question remains which of these analogues qualifies as most suitable. To answer this question, current novel drug agents used in Alzheimer’s research and current medications approved for Alzheimer’s disease management need to be compared to retinoic acid’s properties to assess where these drugs have succeeded and/or failed, and whether retinoic acid is able to correct the aspects that fell short. This will help formulate an approach to what a novel drug candidate should necessarily include. And finally, identifying which of the retinoids fits into this frame would deliver an adequate answer to the question.

Until now, Alzheimer’s disease therapies have focused on two aspects in their approach. These aspects are symptomatic management (therapies that correct neurotransmitter disturbances and psychoactive drugs treating psychiatric disturbances), and disease-modifying therapies aiming towards stopping or modifying the disease progression. Regarding symptomatic therapies, three cholinesterase inhibitors are approved for treatment of mild to moderate Alzheimer’s disease which are donepezil, rivastigmine, and galantamine. The rationale for using these drugs for early Alzheimer’s disease is based on the cholinergic hypothesis of Alzheimer’s disease which proposes that the cholinergic systems in the basal forebrain are altered in the early stages of the disease resulting in loss of acetylcholine neurons, thereby losing enzymatic functions for acetylcholine synthesis and degradation, ultimately resulting in memory loss and decline of other cognitive (as loss of memory) and noncognitive functions (as loss of gross and fine motor functions) that occur in Alzheimer’s disease. By inhibiting cholinesterase, prevention of acetylcholine degradation aims to manage this pathophysiological process. These drugs delay the decline in cognitive function in Alzheimer’s disease by six to 12 months [37].

For severe Alzheimer’s disease symptomatic management, memantine is an approved drug that is an uncompetitive, moderate-affinity N-methyl-D-aspartate (NMDA) antagonist. This drug blocks NDMA receptors and results in the correction of glutamatergic neurotransmission in Alzheimer’s disease [37] which is believed to be hyperactive causing nerve damage by excessive neurotransmitter stimulation also known as excitotoxicity [38]. At six months of usage for moderate and severe Alzheimer’s disease, memantine showed a mild improvement in cognitive symptoms [39]. Therefore, the use of a combination therapy of memantine and donepezil has been proposed and showed a significant improvement in cognitive function, language, and daily life activities in patients with moderate and severe Alzheimer’s disease, but not in patients with mild Alzheimer’s disease [37].

Psychiatric symptoms (such as psychosis, anxiety, depression, aggression, and apathy) are a common occurrence in all stages of Alzheimer’s disease. Therefore, symptomatic therapies aimed to improve these symptoms are used, but do not halt or reverse the disease progression and are of little benefit for cognitive symptoms such as loss of memory. Examples of drugs used for this purpose are serotonin reuptake inhibitors, selective noradrenalin and serotonin inhibitors, atypical antipsychotic agents, and benzodiazepines [37].

At this point, it becomes evident that the future in managing Alzheimer’s disease needs more than mere symptomatic management that eventually fails to halt disease progression which overrides most beneficial effects of these drugs in controlling the symptoms. Hence, disease modifying therapies have been researched and a number of novel agents proposed that aim to halt or reverse disease progression. Anti-amyloid aggregation agents are compounds that inhibit Aβ deposition and thereby reduce the burden of this pathological hallmark. Examples of these compounds that have been used in clinical trials are colostrinin, scyllo-inositol, and tramiprosate. The first two produced disappointing results after phase II trials. Colostrinin, a proline-rich polypeptide complex derived from sheep colostrum, has failed in reproducibility of its modest symptomatic improvement. Scyllo-inositol, a stereoisomer of inositol, has failed to produce any beneficial effects at all [37]. Tramiprosate, a glycosaminoglycan 3-amino-1-propaneosulfonic acid, was the only anti-amyloid aggregation agent qualifying for phase III trials. The results have been disappointing as analyses did not reveal statistically significant differences between groups in a double-blind, placebo-controlled, randomized trial [40]. Moreover, it has been shown that tramiprosate promotes tau aggregation [41], which might partly explain the poor results of the phase III trial.

Selective amyloid β42-lowering agents are a class of disease modifying drugs that reduce amyloid β40 and amyloid β42 production by selectively inhibiting β and γ secretase, or by selectively increasing α-secretase cleavage [42]. CTS-21166 is a β-secretase inhibitor that entered a phase I clinical trial in 2008 conducted by CoMentis and produced promising results as it dose-dependently lowered human plasma Aβ by up to 80% [37]. After that, Astellas Pharma partnered with CoMentis, providing an upfront 80 million US dollars in addition to an eligibility for up to 660 million US dollars in payments linked to development milestones. In 2014, however, Astellas terminated this partnership based on research outcomes that have not been shared publicly. CoMentis classified its CTS-21166 status as confidential since then [43]. The disappointing outcome might be attributed to a number of factors that may offer problematic challenges regarding targeting BACE1. First, mechanism-based toxicities as abnormalities in adult neurogenesis and axon targeting can arise by inhibiting BACE1 as it is an important physiological enzyme, especially that therapy would be chronic and lifelong [44]. Second, due to BACE1’s relatively large molecular size, challenges related to targeting BACE1 and crossing the blood-brain barrier might result in low blood-brain barrier permeability and lead to a reduction in efficacy [37]. Third, it is still uncertain and remains a matter of ongoing research as to when the administration of BACE1 inhibitors will provide optimal results, with more recent results indicating a more prophylactic benefit at early stages of Alzheimer’s disease [44].

More examples of disease-modifying therapies include selective γ-secretase inhibitors as LY-450139 which entered two phase III trials and were both halted due to adverse reactions in the patient groups that received the drug compared to the control placebo group. Moreover, metal-protein-attenuating compounds as PBT2 are a class of drugs interfering with zinc and copper and aim to inhibit the Cu2+ and Zn2+-mediated toxic oligomerization of Aβ. Till now, phase II trials have been conducted with promising results, but large scale phase III trials are yet to be performed [37]. And finally, tau aggregation inhibitors have been one of the most anticipated disease-modifying therapies for Alzheimer’s disease, but have unfortunately failed to deliver its promises with its most recent example, TRx0237, that entered three phase III trials and failed to slow cognitive or functional decline in people with mild to moderate Alzheimer’s disease compared to the placebo groups [45].

Because many drug candidates showed disappointing results, a set of target goals or criteria proposed by Fukasawa et al. outline the main aims of novel candidate disease-modifying drugs, and may also explain why many of the mentioned therapies failed to deliver due to not meeting these criteria [36]. These criteria are expected to be met in preclinical animal models before a drug candidate should enter clinical testing: first, suppression of subacute accumulation of Aβ; second, suppression of brain inflammation by administration of LPS or other agents that produce the same effect; third, functional and histological regeneration of cholinergic and/or glutaminergic nerve destruction by elevation of acetylcholine and choline acetyltransferase levels; fourth, stimulation of impaired nerve regeneration and neurite growth; fifth, improvement of cognition and learning; and finally, tolerability during prolonged use in both animal and human models [36]. Based on this, tamibarotene has been suggested by the authors to be an optimal candidate that meets these criteria. Its chemical stability (compared to ATRA) offers a higher bioavailability and half life, as well as reduced toxic side. Moreover, its properties as an RAR agonist with high specificity for RARα and RARβ grant this compound a wide range of actions on different pathways in Alzheimer’s disease as its transcriptional control of multiple target genes affecting Alzheimer’s disease, reduction of Aβ deposition, suppression of inflammation by inhibition of interferon γ, IL-6, and T helper cell 17 differentiation and inflammatory functions, differentiation of adult forebrain neural progenitor cells into neurons, increased neurite outgrowth, increased acetylcholine levels, behavioral and cognitive improvements in preclinical animal models, and finally, studies on tolerability and prolonged use have been performed as tamibarotene is an approved drug in Japan for the treatment of acute promyelocytic leukemia [36]. All this qualifies tamibarotene as a more comprehensive drug candidate which targets more than one pathophysiological pathway than previously mentioned novel drug candidates which are more selective in their approach and affect one pathway at a time. On this basis, a phase II interventional, randomized, placebo-controlled study is being conducted in Japan for the verification of safety and efficiency of tamibarotene for the treatment of Alzheimer's disease, with the primary outcome measures being the change in Alzheimer’s disease assessment scale, symptoms reports, and laboratory data [46,47].

## Conclusions

Retinoic acid and its derivatives, retinoids, affect Alzheimer’s disease in a number of ways. By binding to the nuclear receptors RAR and RXR, they form complexes that function as ligand-dependent transcriptional regulators that bind to specific RAREs present on the promoter regions of target genes that influence pathophysiological pathways of Alzheimer’s disease, such as SOD-1, SOD-2, ABCA1, PLA2, PLC, PSEN1, PSEN2, BACE1, and MAPT. Pharmacologically, retinoids are unstable compounds that rapidly oxidize due to the presence of conjugated double bonds, explaining the short half-life of ATRA and 13-cis RA. This aspect has been tackled by the development of various synthetic analogues which exhibit higher chemical stability with concurrent higher half-lives. The most prominent example of these analogues is tamibarotene. Most other drugs aside from tamibarotene that target pathways of Alzheimer's disease fall short and partly or fully fail to deliver their promised results. It remains a matter of controversy why the results have been disappointing, but it is proposed that one of the main reasons is that these drugs target single pathways in Alzheimer’s disease and do not target multiple pathways at once. This problem, however, is theoretically solved by tamibarotene as it targets multiple pathways at once as transcriptional control of target genes, reduction of Aβ deposition, suppression of inflammation, differentiation of adult forebrain neural progenitor cells into neurons, increased neurite outgrowth, increased acetylcholine levels, and improved tolerability with less adverse reactions compared to the drugs mentioned above, making it a novel drug candidate for Alzheimer’s disease. 
